# Selection of reference genes for normalization of quantitative real-time PCR in organ culture of the rat and rabbit intervertebral disc

**DOI:** 10.1186/1756-0500-4-162

**Published:** 2011-05-26

**Authors:** Dongrim Seol, Hyeonghun Choe, Hongjun Zheng, Keewoong Jang, Prem S Ramakrishnan, Tae-Hong Lim, James A Martin

**Affiliations:** 1 Department of Orthopedics and Rehabilitation, University of Iowa, 1182 ML, Iowa city, IA 52242, USA; 2 Department of Biomedical Engineering, University of Iowa, 1402 SC, Iowa city, IA 52242, USA

## Abstract

**Background:**

The accuracy of quantitative real-time RT-PCR (qRT-PCR) is often influenced by experimental artifacts, resulting in erroneous expression profiles of target genes. The practice of employing normalization using a reference gene significantly improves reliability and its applicability to molecular biology. However, selection of an ideal reference gene(s) is of critical importance to discern meaningful results. The aim of this study was to evaluate the stability of seven potential reference genes (Actb, GAPDH, 18S rRNA, CycA, Hprt1, Ywhaz, and Pgk1) and identify most stable gene(s) for application in tissue culture research using the rat and rabbit intervertebral disc (IVD).

**Findings:**

*In vitro*, four genes (Hprt1, CycA, GAPDH, and 18S rRNA) in rat IVD tissue and five genes (CycA, Hprt1, Actb, Pgk1, and Ywhaz) in rabbit IVD tissue were determined as most stable for up to 14 days in culture. Pair-wise variation analysis indicated that combination of Hprt1 and CycA in rat and the combination of Hprt1, CycA, and Actb in rabbit may most stable reference gene candidates for IVD tissue culture.

**Conclusions:**

Our results indicate that Hprt1 and CycA are the most stable reference gene candidates for rat and rabbit IVD culture studies. In rabbit IVD, Actb could be an additional gene employed in conjunction with Hprt1 and CycA. Selection of optimal reference gene candidate(s) should be a pertinent exercise before employment of PCR outcome measures for biomedical research.

## Background

Quantitative real-time RT-PCR (qRT-PCR) is a powerful tool for detection and quantification of gene expression owing to its high sensitivity, specificity and reproducibility [1]. However, the relevance and magnitude of absolute measures obtained from qRT-PCR are subject to inherent sample variations that usually lead to statistical uncertainty. Such outcomes may also lead to inferences that may be biologically obscure [2]. To this end, an appropriate normalization strategy is employed for reliable data interpretation and the most common method is the use of an internal reference or housekeeping gene [3]. A reference gene is weakly regulated in experimental conditions of interest along with comparable expression characteristics to target genes. Thus, selection process of an ideal reference gene is critical for applicability of PCR in research. However, commonly used reference genes for tissue and cell-based PCR normalization such as glyceraldehydes-3-phosphate dehydrogenase (GAPDH), β-actin (Actb) and 18S ribosomal RNA (18S rRNA) are frequently applied without appropriate validation for their gene expression stability [4-6].

Gene expression analyses are used as one of the major contemporary research tools in understanding the pathology of intervertebral disc (IVD) degeneration. Clinically termed as "Degenerative Disc Disease (DDD)", the condition is believed to be a significant source of low back pain [7,8]. The clinical significance of understanding the onset and progress of DDD is well documented and there is increasing need to establish relevant experimental models to study this disease. Numerous studies on molecular level changes in disc biology have been reported by normalization using GAPDH [9-12] and Actb [13-15] without validation for their stability. Although the assumption of certain genes being constitutively expressed may be valid in certain cases, this assumption cannot be taken for granted under rapidly changing conditions such as growth, remodeling and disease.

The aim of this research was to evaluate the stability of seven potential reference genes (Actb, GAPDH, 18S rRNA, CycA, Hprt1, Ywhaz, and Pgk1) and select the most stable genes or a combination of stable genes for the purpose of normalization in IVD gene expression studying of rat and rabbit organ culture. The stability of selected reference genes under different experimental culture periods and species was analyzed using geNorm [6], NormFinder [5] and BestKeeper [16].

## Results

### Quantitative real-time RT-PCR

Seven candidate reference genes were selected from commonly used housekeeping genes which have different biologic function (Table 1). Their primer information was summarized in Table 2.

**Table 1:** Description of candidate reference genes for qRT-PCR

Abbreviation	Gene	Function
**Actb**	β-actin	Cytoskeletal structural protein
**GAPDH**	Glyceraldehydes-3-phosphate dehydrogenase	Carbohydrate metabolism
**18S rRNA**	18S ribosomal RNA	Cytosolic small ribosomal subunit, translation
**CycA**	Cyclophilin A	Catalyzes the cis-trans isomerization of proline imidic peptide bonds in oligopeptides, accelerating folding
**Hprt1**	Hypoxanthine phosphoribosyltransferase 1	Metabolic salvage of purines in mammals
**Ywhaz**	Tyrosine 3-monooxygenase	Signal transduction by binding to phosphorilated serine residue on a variety of signaling molecules
**Pgk1**	Phosphoglycerate kinase 1	Transferase enzyme in the glycolysis

**Table 2:** Primer information of reference genes for qRT-PCR

	Sprague-Dawley rat		New Zealand White Rabbit	
	
	Forward (5'-3') Reverse (5'-3')	Product size (bp) [Ref]	Forward (5'-3') Reverse (5'-3')	Product size (bp) [Ref]
**Actb**	AGGCCAACCGTGAAAAGATG ACCAGAGGCATACAGGGACAA	101 [33]	CTGGAACGGTGAAGGTGACA CGGCCACATTGCAGAACTTT	73 [D]
**GAPDH**	GCAAGAGAGAGGCCCTCAG TGTGAGGGAGATGCTCAGTG	74 [18]	GGGTGGTGGACCTCATGGT CGGTGGTTTGAGGGCTCTTA	58 [D]
**18S rRNA**	ACGGACCAGAGCGAAAGCAT TGTCAATCCTGTCCGTGTCC	310 [19]	TCGGCATTCGAACGTATGC ACCCGTGGTCACCATGGTA	56 [D]
**CycA**	TATCTGCACTGCCAAGACTGAGTG CTTCTTGCTGGTCTTGCCATTCC	126 [20]	CCAACGGCTCCCAGTTCTT ACGTGCTTGCCGTCCAA	61 [D]
**Hprt1**	TGTTTGTGTCATCAGCGAAAGTG ATTCAACTTGCCGCTGTCTTTTA	66 [D]	GCAGACCTTGCTTTCCTTGGT GCAGGCTTGCGACCTTGAC	63 [D]
**Ywhaz**	TTGAGCAGAAGACGGAAGGT GAAGCATTGGGGATCAAGAA	136 [19]	GGTCTGGCCCTTAACTTCTCTGTGTTCTA GCGTGCTGTCTTTGTATGATTCTTCACTT	142 [28]
**Pgk1**	ATGCAAAGACTGGCCAAGCTAC AGCCACAGCCTCAGCATATTTC	104 [20]	TGTTGGTCGGGCGAAGCAG CAGTGTCTCCACCGCCGATG	149 [28]

[D] designed primer

**Figure 1:**
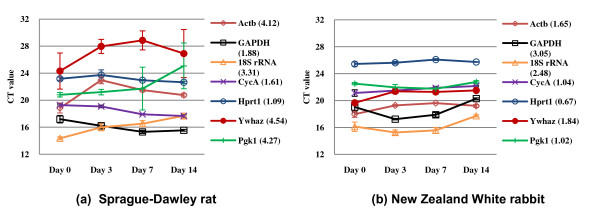
**Cycle threshold (C_T_) values of candidate reference genes in intervertebral disc organ culture of Sprague-Dawley rat (a) and New Zealand White rabbit (b)**. C_T _values represent mean ± S.D. from three biological replicates. C_T _rang calculated from maximum and minimum is shown between parentheses in each reference genes.

All RNA samples were examined for their purity. The absorbance ratio at A260/A280 nm of all samples was ranged from 1.86 to 2.09, indicating all the samples were pure during the RNA extraction procedure. For all the candidate reference genes, the melt curve analyses of PCR reactions were performed. The specificity and integrity of the products were confirmed by the presence of a single peak in dissociation curve (additional file 1). The standard curves were made by serial dilutions to determine PCR efficiencies. The qRT-PCR efficiency (E) of each primer pair was ranged from 1.901 to 2.141 (90.1% to 114.1%) with linear correlation coefficient (R^2^), making all assays suitable for quantitative analysis (Table 3). Figure 1 represents C_T _values of candidate reference genes from IVD organ culture of SD rat and NZW rabbit. C_T _values of reference genes obtained by the rat IVD were more variable with higher standard deviation than those of the rabbit IVD. To quantify the variation, C_T _range was calculated by maximum and minimum values through four harvesting time points. Hprt1 was shown the most invariable expression between all samples for both rat (1.09) and rabbit (0.67). In rat, CycA (1.61) and GAPDH (1.88) were ranked as second and third gene, respectively. In rabbit, on the other hand, Pgk1 (1.02) and CycA (1.04) were ranked as second and third genes, respectively. This implies that Hprt1, CycA, and GAPDH in rat and Hprt1, Pgk1, and CycA are more potential reference genes compared to the others.

**Table 3:** Standard curve parameters for candidate reference genes

	SD rat			NZW rabbit		
	
Genes	Slope	Efficiency (E) [%]	**Coefficient (R**^**2**^**)**	Slope	Efficiency (E)	**Coefficient (R**^**2**^**)**
Actb	-3.024	2.141 [114.1]	0.992	-3.363	1.983 [98.3]	0.999
GAPDH	-3.222	2.043 [104.3]	0.995	-3.388	1.973 [97.3]	0.997
18S rRNA	-3.253	2.030 [103.0]	0.982	-3.584	1.901 [90.1]	0.994
CycA	-3.135	2.084 [108.4]	0.996	-3.091	2.106 [110.6]	0.999
Hprt1	-3.360	1.984 [98.4]	0.991	-3.411	1.964 [96.4]	0.990
Ywhaz	-3.043	2.131 [113.1]	0.994	-3.040	2.133 [113.3]	0.985
Pgk1	-3.078	2.113 [104.6]	0.992	-3.039	2.133 [113.3]	0.986

### geNorm analysis

**Figure 2:**
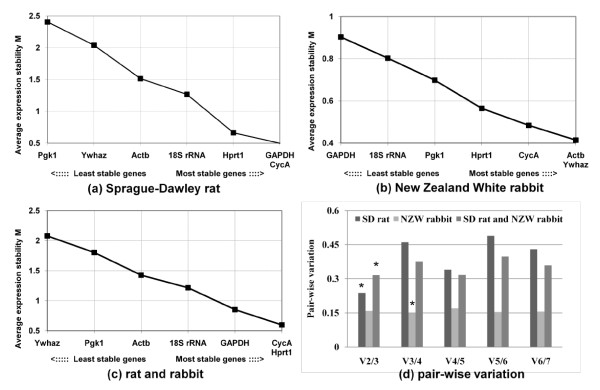
**Ranking of the reference genes and pair-wise variation by geNorm**. Candidate reference genes were ranked according to the average expression stability in intervertebral disc organ culture of Sprague-Dawley rat (a), New Zealand White rabbit (b), and combined species (c). Pair-wise variations (V_n/n+1_) were calculated to determine the minimum number of reference genes. The cutoff value was set at 0.15. The optimal variation is marked with a star (*).

The gene expression stability of seven candidate reference genes over the organ culture of the rat and rabbit IVD was analyzed using the geNorm software applications. The analysis by geNorm showed that only four genes (GAPDH, CycA, Hprt1, and 18S rRNA) in the rat IVD reached a high expression stability with low M values, below the default limit of M = 1.5 [6] (Figure 2a). GAPDH and CycA were identified as the best pair of reference genes. On the other hand, all reference genes showed stable M value (M < 1.5), and Actb and Ywhaz were ranked as the most stable reference genes in rabbit (Figure 2b). CycA and Hprt1 were ranked as third and fourth genes, respectively. Similarly, CycA and Hprt1 were identified to be suitable for normalization in the combined analysis obtained from rat and rabbit (Figure 2c). To determine the optimal number of reference genes necessary for accurate normalization, calculation of the pair-wise variation (V_n/n+1_) was evaluated (Figure 2d). The combination of two reference genes was suitable for normalizing gene expression data in rat and combined species, V_2/3 _(0.238) and V_2/3 _(0.316) respectively, which is close to the cutoff value 0.15. On the other hand, in rabbit samples, all pair-wise variations were close to cutoff value and three reference genes would be sufficient to normalize the target genes.

### NormFinder and BestKeeper analysis

Analysis on the stability of the reference genes using NormFinder and BestKeeper showed different ranking order compared to geNorm analysis (Table 4). NormFinder indicated Hprt1 as the best stable gene in rat and combined species, followed by 18S rRNA, CycA, and GAPDH. All reference genes in rabbit showed better stability value compared to those of rat, and CycA ranked as best control gene with a stability value of 0.227. The BestKeeper calculated variations for all the reference genes based on geometric mean of the C_T _values. Genes with standard deviation (SD) higher than 1 were defined as unstable. Similar to NormFinder, Hprt1 showed the lowest C_T _value variation in rat and combined species, and GAPDH and CycA with lower than 1 (SD) were indicated as good candidate genes. In rabbit, all reference genes except GAPDH showed low SD values and Hprt1 which was second ranking order in NormFinder was calculated to be the most stable gene with a SD value of 0.23.

**Table 4:** Ranking order of candidate reference genes in the intervertebral disc of Sprague-Dawley (SD) rat and New Zealand White (NZW) rabbit

	NormFinder			BestKeeper		
	
Rank	SD rat	NZW rabbit	rat & rabbit	SD rat	NZW rabbit	rat & rabbit
**1**	Hprt1 (0.541)	CycA (0.227)	Hprt1 (0.514)	Hprt1 0.35)	Hprt1 (0.23)	Hprt1 (0.30)
**2**	18S rRNA (0.755)	Hprt1 (0.371)	18S rRNA (0.596)	GAPDH (0.66)	CycA (0.36)	CycA (0.68)
**3**	CycA (0.788)	Pgk1 (0.374)	CycA (0.636)	CycA (0.69)	Pgk1 (0.43)	GAPDH (0.85)
**4**	GAPDH (0.931)	Actb (0.478)	Actb (0.658)	18S rRNA (1.05)	Actb (0.53)	Actb (0.97)
**5**	Actb (0.947)	Ywhaz (0.497)	Ywhaz (0.783)	Actb (1.23)	Ywhaz (0.64)	Pgk1 (1.01)
**6**	Pgk1 (1.417)	18S rRNA (0.511)	Pgk1 (0.808)	Ywhaz (2.22)	18S rRNA (0.90)	Ywhaz (1.12)
**7**	Ywhaz (1.482)	GAPDH (0.699)	GAPDH (0.956)	Pgk1 (2.25)	GAPDH (1.12)	18S rRNA (1.23)

(value); stability in NormFinder and standard deviation in BestKeeper

### Overall ranking order and selection of the best genes

All results obtained from three programs showed different ranking order according to their different calculated algorithm. To find the combination of stable reference genes, we summarized the output results which were M value (geNorm), stability (NormFinder), and standard deviation (BestKeeper) and calculated the average and ranking order in Table 5. Based on the pair-wise variation analysis by geNorm, the number of stable reference genes was applied as two for rat and combined species and three for rabbit. The overall ranking order of SD rat was Hprt1, CycA, GAPDH, 18S rRNA, Actb, Ywhaz, and Pgk1 and the combination of Hprt1 and CycA was finally selected as best control genes. Similarly, the combination of Hprt1 and CycA were determined as the most stable housekeeping genes in analyzing of combined speices. In experiments with rabbit intervertebral disc, however, the most preferred reference genes were CycA, Hprt1, and Actb.

**Table 5:** Overall ranking order of candidate reference genes based on the output values from three programs and selection of the best genes in the intervertebral disc of Sprague-Dawley (SD) rat and New Zealand White (NZW) rabbit

SD rat	Actb	GAPDH	18S rRAN	CycA	Hprt1	Ywhaz	Pgk1	Best genes
**geNorm**	1.515	0.498	1.268	0.498	0.664	2.040	2.406	
**NormFinder**	0.947	0.931	0.755	0.788	0.541	1.482	1.417	**Hprt1 and CycA**
**BestKeeper**	1.23	0.66	1.05	0.69	0.35	2.22	2.25	
**Average**	1.23	0.70	1.02	0.66	0.52	1.91	2.02	
**Rank**	5	3	4	2	1	6	7	

**NZW rat**	**Actb**	**GAPDH**	**18S rRAN**	**CycA**	**Hprt1**	**Ywhaz**	**Pgk1**	**Best genes**

**geNorm**	0.414	0.903	0.803	0.484	0.565	0.414	0.698	
**NormFinder**	0.478	0.699	0.511	0.227	0.371	0.497	0.374	
**BestKeeper**	0.53	1.12	0.90	0.36	0.23	0.64	0.43	**CycA Hprt1 and Actb**
**Average**	0.47	0.91	0.74	0.36	0.39	0.52	0.50	
**Rank**	3	7	6	1	2	5	4	

**rat & rabbit**	**Actb**	**GAPDH**	**18S rRAN**	**CycA**	**Hprt1**	**Ywhaz**	**Pgk1**	**Best genes**

**geNorm**	1.427	0.854	1.217	0.598	0.598	2.081	1.804	
**NormFinder**	0.658	0.956	0.596	0.636	0.514	0.783	0.808	
**BestKeeper**	0.97	0.85	1.23	0.68	0.30	1.12	1.01	**Hprt1 and CycA**
**Average**	1.02	0.89	1.01	0.64	0.47	1.33	1.21	
**Rank**	5	3	4	2	1	7	6	

## Discussion

It has become increasingly clear in recent years that the accuracy of qRT-PCT analysis strongly depends on the choice of the normalization approach. Among current normalization approaches available, the use of housekeeping gene as an internal control is by far the most convenient to compute, provided the biological assumption of the reference gene is fulfilled. Several studies have demonstrated that commonly used reference genes are regulated under various experimental conditions, indicating the requirement of reference gene validation as the first step before meaningful outcomes could be discerned. To further improve sensitivity, normalization with multiple reference genes is also proposed.

Rat and rabbit are preferred models for studying the pathogenesis of disc degeneration. Current knowledge suggests that mechanical stimulation may be a significant contributor to the biology of the IVD. To this end, our working hypotheses states that abnormal mechanical stimulation can induce pertinent biological cascades that may degenerate a healthy normal disc in a temporo-spatial manner. Although *in vivo *mechanical stimulation is possible to a certain extent, understanding the effect of complex biomechanics would be achievable to a greater extent *in vitro *using a whole organ system. Successful realization of a mechanically-active culture system would require validation of the experimental model using well characterized outcome measures. Among several outcomes that need to be verified for the evaluation of our *in vitro *experimental model, expression profiles of genes of interest and validation of reference gene(s) are considered significant requirements.

To determine the most stable genes, data were analyzed using three different applications (geNorm, NormFinder, and BestKeeper) with distinct algorithms for assessing and identifying gene stability. Briefly, GeNorm software identifies stable genes based on the principle that two ideal genes have same expression ratios in all samples. The expression ratio is reported as the stability value 'M' that is essentially an average pair-wise variation of each gene in comparison to other candidate genes. NormFinder utilizes expression variation between inter and intra groups for identifying the ideal reference genes. In other words, the program can calculate not only the overall variation of candidate reference genes but also the variation between subgroups of given sample set. BestKeeper calculates reference gene's standard deviation (SD) based on raw C_T _values regardless of sample's efficiency. Gene with the lowest SD is considered the most stable gene. We applied these three programs for complementary analyses. As expected, ranking data reported by the programs differed considerably in terms of magnitude of the ranking parameter and order of ranking. Surprisingly (although with varying rank order), four genes (Hprt1, CycA, GAPDH, and 18S rRNA) in rat IVD tissue and five genes (CycA, Hprt1, Actb, Pgk1, and Ywhaz) in rabbit IVD tissue were presented as available reference genes. To determine a consolidated rank ordering of reference genes using data from all the output, we decided to average the outcome measures of the three programs for each reference gene since the three algorithms determined the gene ranking based on the function of decreasing value of their outcome measures. Previously, Axtner et al. [17] identified the best combination of genes by mean rank derived from the three programs. We employed a different strategy by using the mean of three outcome measures and ranked the genes of interest as a function of their means with the lower means ranking better than their counterparts. We found this strategy to be more appropriate for the purpose of mathematical clarity.

In conjunction with GAPDH, Actb and 18S rRNA, our selection of other potential reference genes were based on demonstrated results of stability in previous investigations Xing et al. [18] demonstrated Hprt1 to be the best reference gene in rat partial hepatectomy model, which is experimental model for the study of liver regeneration. On the other hand, Ywhaz and CycA were the most stable genes in the brain tissue and asphyxia cardiac arrest model, respectively [19,20]. In the current study, combination of Hprt1 and CycA were identified to be suitable for normalization for rat and rabbit IVD culture studies. Although expression stability of reference genes may be influenced by multiple factors, our findings suggest that experimental conditions have a significant effect in this *in vitro *model.

GAPDH has been successfully employed as a reference gene in IVD organ culture studies [21,22] whereas our study indicates otherwise. The instability of GAPDH in our culture model could partly be the result of oxygen tension gradients in the IVD [23] that may result in regulation of HIF-1α [24]. Actb and 18S rRNA were also observed to be regulated in our culture model despite successful application in previous studies [25-27]. We analyzed matrix metalloproteinase-3 (MMP-3) expression which plays a major role in the disc degeneration process using Actb and combination of Hprt1 and CycA. MMP-3 expression normalized by Hprt1 and CycA showed 3.8-fold up-regulation at day 3, while Actb induced 73.7-fold up-regulation in the rat IVD tissue (additional file 2). Our comparative analysis emphasizes the critical requirement of reliable reference gene(s) to avoid erroneous and misrepresented results.

The delta-delta C_T _algorithm is a convenient and standard method to analyze the relative changes in gene expression. This method requires the C_T _values for a reference gene(s) to be reliably lower of that of a target gene. However, C_T _values of rabbit Hprt1 were relatively higher than those of other optimal reference genes (CycA and Actb) in this study. Although the C_T _values can be reduced by using increased amount of PCR templates, we first need to confirm the range of C_T _values of target genes for accurate analysis of relative quantification.

Accurate normalization determines the sensitivity and reproducibility of a PCR measure making the selection process of a reference gene a very crucial step in validating the gene expression tool. It is also suggested by previous investigations that single control normalization may still lead to erroneous results, urging the need to use two or more reference genes to improve sensitivity and also maintain low expression variation [6]. It is also generally advised to maintain Ct values < 30 so that the initial abundance of the target gene is considered biologically consequential. We realized the significance of multiple reference genes and have reported two most stable genes for each individual small animal species. We believe the recommendations of this study are applicable for future investigations in IVD biology that use rat and rabbit disc tissues. Also, our inference is solely concerned with the experimental conditions stated in this investigation.

## Conclusions

We evaluated the stability of seven potential reference genes (Actb, GAPDH, 18S rRNA, CycA, Hprt1, Ywhaz, and Pgk1) and attempted to identify the most stable control gene (s) for normalization of qRT-PCR data from rat and rabbit organ culture. Using geNorm, NormFinder, and BestKeeper, we determined that Hprt1 and CycA are ideal reference gene candidates in both the species of interest. Actb was also found suitable for normalization in the rabbit IVD whereas its stability is questionable in the rat model. GAPDH was found to be unsuitable for normalization in both rat and rabbit IVDs under our experimental constraints. Data presented in this work is the first of its kind focusing on the intervertebral disc and may facilitate improvement in reliability and sensitivity of qRT-PCR for IVD organ culture studies.

## Methods

### Intervertebral disc (IVD) organ culture

Young adult male Sprague-Dawley (SD) rats that were 9-weeks (280 g) old and New Zealand White (NZW) rabbits that were 18-weeks (3.6 kg) old were obtained from Harlan Sprague Dawley, Inc. (Indianapolis, IN, USA). The animals were used in accordance with a protocol approved by the University of Iowa Animal Care and Use Facilities. Under sterile condition, animals were sacrificed and lumbar IVD motion segments were dissected from consecutive levels (L1-L6). Posterior elements and soft tissues were removed and cultured in Dulbecco's modified Eagle medium (DMEM) supplemented with 14% fetal bovine serum (FBS), 50 ㎍/㎖ L-ascorbate, 100 U/㎖ penicillin, 100 ㎍/㎖ streptomycin, and 2.5 ㎍/㎖ Fungizone. After 0, 3, 7, and 14 days under standard culture conditions (37°C, 5% CO_2_), randomly harvested IVDs were isolated from adjacent vertebral bodies and immediately frozen in liquid nitrogen. Three IVDs were pooled together and used for RNA isolation.

### RNA isolation

Samples were homogenized with TRIzol^® ^reagent (Invitrogen™ Life Technologies, Carlsbad, CA, USA) and total RNA was extracted by the homogenized tissues using the RNeasy Mini Kit (Qiagen, Valencia, CA, USA) according to the manufacture's instructions. Total RNA was quantified using a NanoDrop^® ^ND-1000 UV-Vis Spectrophotometer (Thermo Scientific, Waltham, MA, USA) at a 260 nm wavelength.

### Candidate reference genes and primers for qRT-PCT

Candidate reference genes selected were classical reference genes which are most commonly used as internal control for gene expression studies (Actb, GAPDH, and 18S rRNA) and the others (CycA, Hprt1, Ywhaz, and Pgk1) based on previous reports [19,20,28]. Most primer information was obtained from previously published primer sequences. One rat (Hprt1) and five rabbit primers (Actb, GAPDH, 18S rRNA, CycA, and Hprt1) were designed using the Primer Express^® ^3.0 software (Applied Biosystems, Foster City, CA, USA) based on the sequences in the database [29].

### Quantitative real-time RT-PCR (qRT-PCR)

qRT-PCR was performed with the SuperScriptTM III Platinum^® ^SYBR^® ^Green One-Step qRT-PCR kit (Invitrogen™ Life Technologies) following the instructions with slight modification. For each sample, 50 ng total RNA was used in the assay for all reference genes except rat GAPDH (25 ng), rat 18S (1 ng), and rabbit Actb (25 ng) and all samples were run in triplicate on a 96-well optical reaction plate with the ABI PRISM 7700 Sequence Detection System (Applied Biosystems) with a Sequence Detection System (SDS) software version 2.3. The PCR Reactions were prepared in a total volume of 25 ㎕ containing 1 ㎕ diluted RNA, 0.5 ㎕ forward and reverse primer (10 μM), 12.5 ㎕ 2X SYBR^® ^Green Reaction Mix, and 10 ㎕ RNase-free water. The conditions for the PCR were as follows: reverse transcription at 50°C for 3 min, DNA polymerase activation and RT enzyme inactivation at 95°C for 5 min, followed by 40 cycles of denaturation at 95°C for 15 sec, primer annealing at 60°C for 30 sec, elongation at 40°C for 1 min. The quantification values were obtained from the threshold cycle (C_T_) number at which the increase in signal associated with an exponential growth of PCR products using SDS software. At the end of the PCR reactions, amplification specificity was confirmed by analyzing dissociation curve. To calculate PCR efficiency for each gene, six points of 2-fold serial dilution were used to build standard curve.

### Data analysis

To calculate the C_T _values, the fluorescence threshold was manually set to 1 in SDS software and the results were directly imported into Microsoft Excel for BestKeeper (version 1.0) [30] data input. C_T _values above 30 were excluded in this study. PCR efficiencies (E) were calculated from the slope of each standard curve with the equation,

Relative quantities (Q) was then calculated from the C_T _values and efficiencies for geNorm (version 3.5) [31] and NormFinder (version 19.0) [32] data input with the equation,

The gene expression stability was ranked from each program and the most stable reference gene or combination genes were calculated.

## Competing interests

The authors declare that they have no competing interests.

## Authors' contributions

DS harvested the intervertebral disc, performed qRT-PCR experiments and wrote the paper. HC designed primers and performed qRT-PCR. KJ participated in the data analysis. HZ advised all qRT-PCR process. TL and JAM supervised the study design and PSR helped to draft the manuscript. All authors read and approved the final manuscript.

## Supplementary Material

Additional file 1 **Melting curve analysis**. Melting curve analysis of 7 candidate reference genes using ABI PRISM 7700 Sequence Detection System (Applied Biosystems) with a Sequence Detection System (SDS) software version 2.3. Click here for file

Additional file 2 **MMP-3 gene expression in the rat intervertebral disc**. Relative expression level of MMP-3 in the rat intervertebral disc normalized by common used reference genes (Actb, GAPDH and 18S rRNA) and an optimal combination of reference genes (Hprt1 and CycA). Click here for file
