# Supplementary material for: Selection of reference genes for normalization of quantitative real-time PCR in organ culture of the rat and rabbit intervertebral disc
Source: BMC Res Notes. 2011 May 26;4:162. doi: 10.1186/1756-0500-4-162 (PMC3118343; doi:10.1186/1756-0500-4-162)
Supplement: Additional file 1 — Melting curve analysis. Melting curve analysis of 7 candidate reference genes using ABI PRISM 7700 Sequence Detection System (Applied Biosystems) with a Sequence Detection System (SDS) software version 2.3. [file 1756-0500-4-162-S1.PDF]

Rat Actb

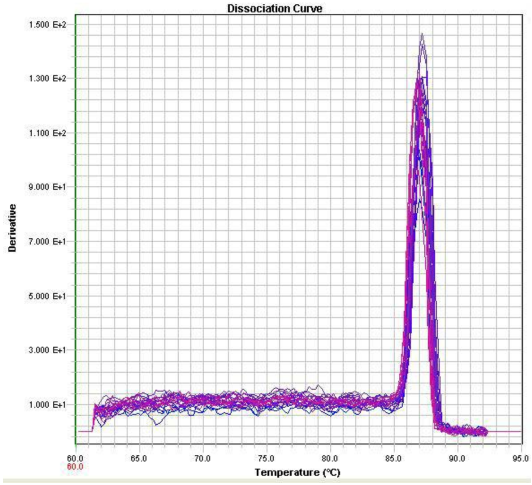

Rat GAPDH

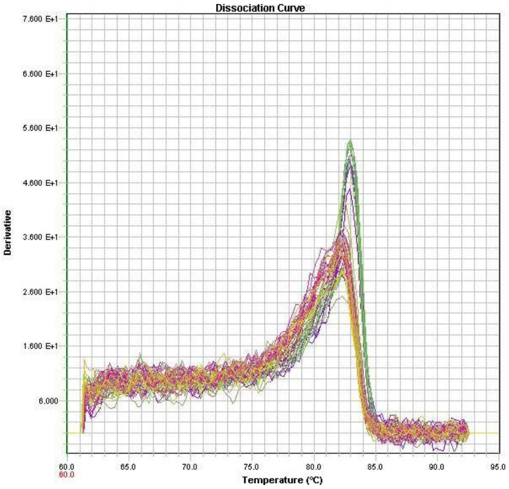

Rat 18S rRNA

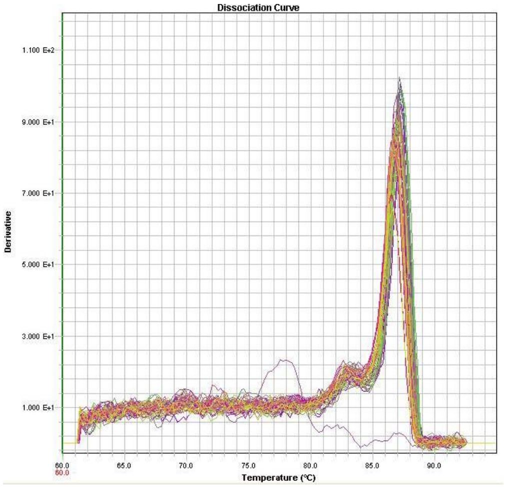

Rat CycA

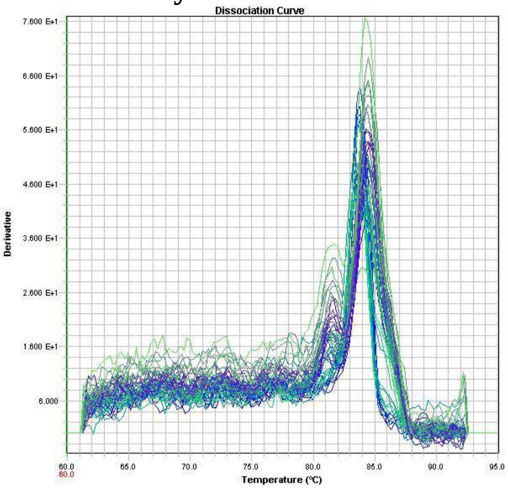

Rat Hprt1

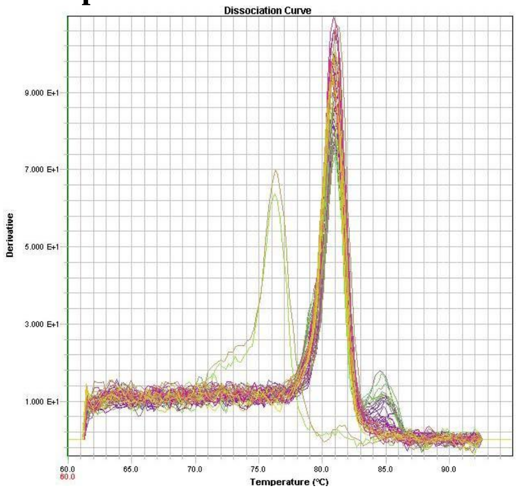

Rat Ywhaz

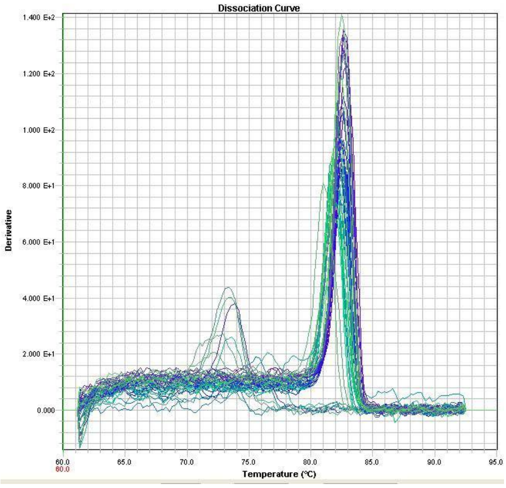

Rat Pgk1

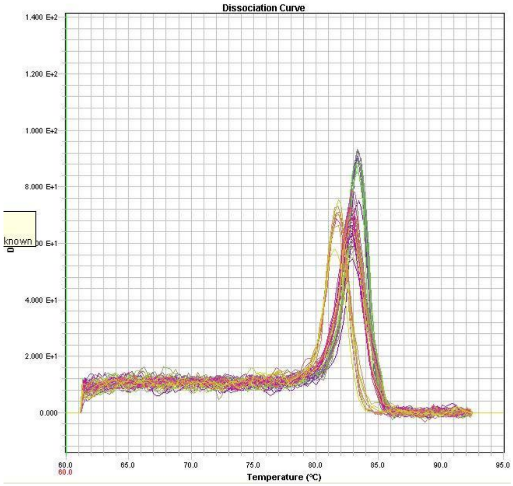

Rabbit Actb **Rabbit GAPDH**

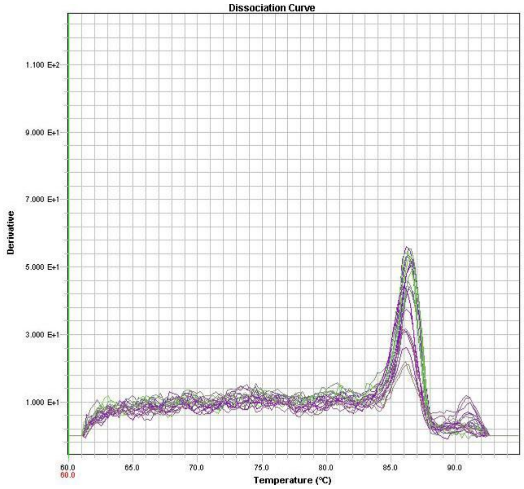

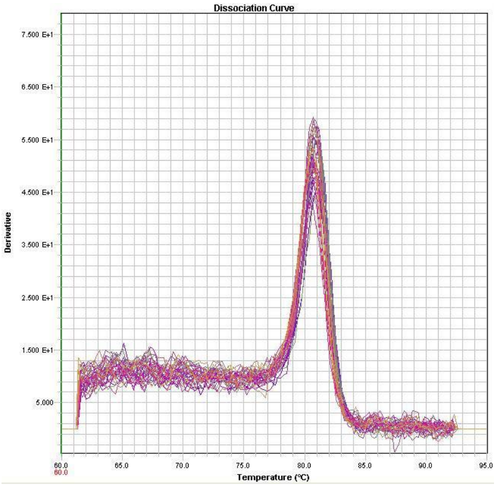

Rabbit 18S rRNA **Rabbit CycA**

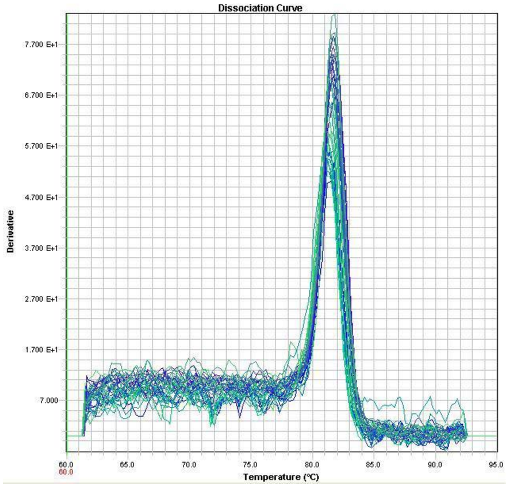

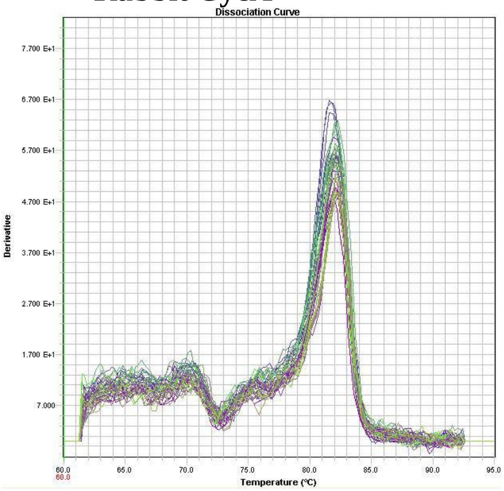

Rabbit Hprt1 **Rabbit Ywhaz**

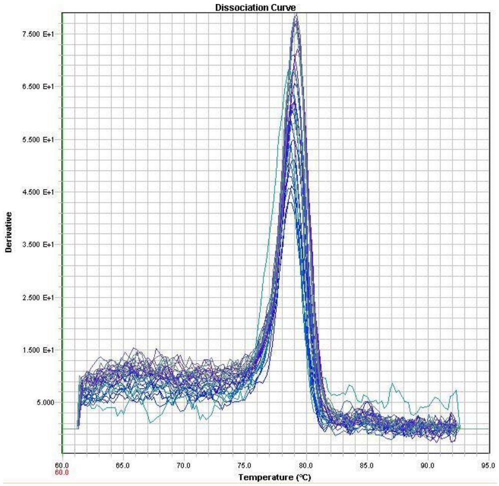
Dissociation Curve
Derivative
7.500 E+1
6.500 E+1
5.500 E+1
4.500 E+1
3.500 E+1
2.500 E+1
1.500 E+1
5.000
60.0
65.0
70.0
75.0
80.0
85.0
90.0
95.0
60.0
Temperature (°C)

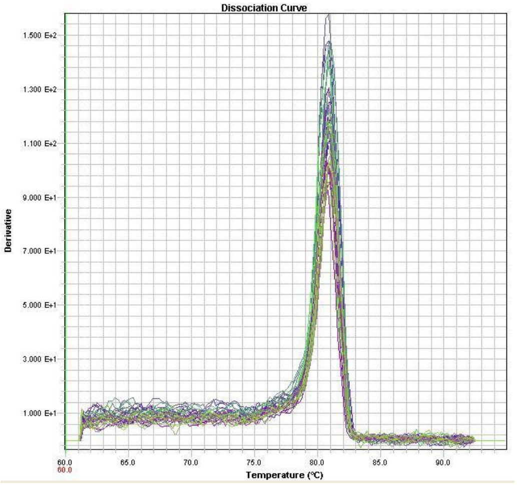
Dissociation Curve
Derivative
1.500 E+2
1.300 E+2
1.100 E+2
9.000 E+1
7.000 E+1
5.000 E+1
3.000 E+1
1.000 E+1
60.0
65.0
70.0
75.0
80.0
85.0
90.0
60.0
Temperature (°C)

Rabbit Pgk1

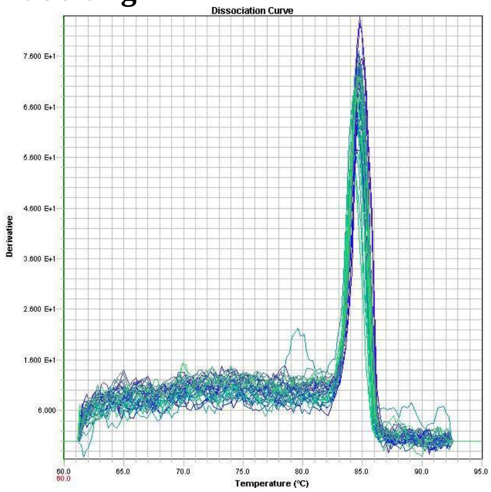
Dissociation Curve
Derivative
7.600 E+1
6.600 E+1
5.600 E+1
4.600 E+1
3.600 E+1
2.600 E+1
1.600 E+1
6.000
60.0
65.0
70.0
75.0
80.0
85.0
90.0
95.0
60.0
Temperature (°C)
