# Supplementary figures and images for: Selection of reference genes for normalization of quantitative real-time PCR in organ culture of the rat and rabbit intervertebral disc
Source: BMC Res Notes. 2011 May 26;4:162. doi: 10.1186/1756-0500-4-162 (PMC3118343; doi:10.1186/1756-0500-4-162)

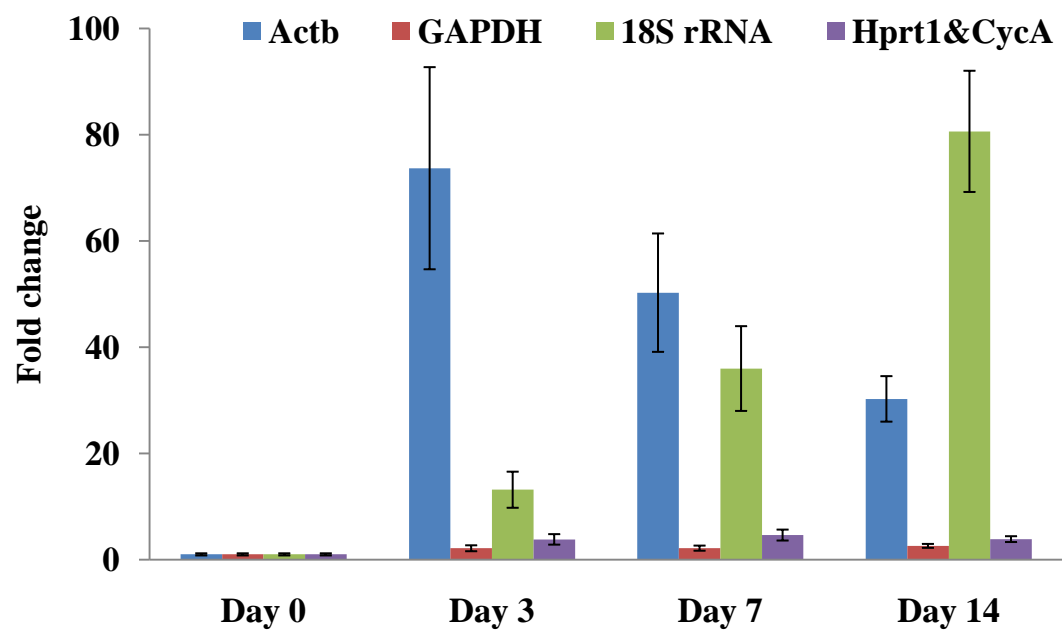

Supplement: Additional file 2 — MMP-3 gene expression in the rat intervertebral disc. Relative expression level of MMP-3 in the rat intervertebral disc normalized by common used reference genes (Actb, GAPDH and 18S rRNA) and an optimal combination of reference genes (Hprt1 and CycA). [file 1756-0500-4-162-S2.PDF]
